# 

**Published:** 1895-04

**Authors:** 


### CONTENTS.

ORIGINAL ARTICLES:

Address at the Commencement of the New York College of Veterinary Surgeons. By PROFESSOR JAMES LAW . . . 201

The Anatomy of the Large American Fluke (*Fasciola Magna*) and a Comparison with Other Species of the Genus Fasciola, S.ST. By CHAS. WARDELL STILES, PH.D. . . . 213

Enchondroma in the Bitch. By C. A. BOUTELLE . . . 222

Antitoxin. I. The Production of Diphtheria Toxin. By WILLIAM HALLOCK PARK, M.D. . . . 228

II. The Production of Diphtheria Antitoxin. By H. D. GILL, V.S. . . . 229

III. Therapeutic Use of Antitoxin. By J. H. HUDDLESTON, M.D. . . . 233

COMMENCEMENT EXERCISES:

Ontario Veterinary College . . . 236

Kansas City Veterinary College . . . 240

American Veterinary College . . . 241

New York College of Veterinary Surgeons . . . 242

PERSONAL . . . 244

EDITORIALS . . . 246

OBITUARY—John Gamgee . . . 247

REVIEWS—Friedberger and Fröhner's *Pathology and Therapeutics of the Domestic Animals—Antisepsis and Antiseptics* . . . 247, 248

CORRESPONDENCE . . . 248

REPORTS OF CASES . . . 249

SELECTION:

Guernsey Breeders' Association. . . . 250

SOCIETY PROCEEDINGS:

California State Veterinary Medical Association . . . 253

Keystone Veterinary Medical Association . . . 257

Wisconsin Society of Veterinary Graduates . . . 258

Missouri Valley Veterinary Association . . . 259

Annual Meeting of the American Veterinary College Alumni Association . . . 260

LEGISLATION—ITEMS OF INTEREST—POINTS WORTH KNOWING . . . 261–268

